# Improved molecular detection of *Babesia* infections in animals using a novel quantitative real-time PCR diagnostic assay targeting mitochondrial DNA

**DOI:** 10.1186/s13071-017-2064-1

**Published:** 2017-03-07

**Authors:** Barbara A. Qurollo, Nikole R. Archer, Megan E. Schreeg, Henry S. Marr, Adam J. Birkenheuer, Kaitlin N. Haney, Brittany S. Thomas, Edward B. Breitschwerdt

**Affiliations:** Vector-borne Disease Diagnostic Laboratory, Comparative Medicine Institute, College of Veterinary Medicine, North Carolina State University, Raleigh, NC USA

**Keywords:** Canine babesiosis, *Babesia*, Mitochondrial DNA, Quantitative PCR

## Abstract

**Background:**

Babesiosis is a protozoal, tick transmitted disease found worldwide in humans, wildlife and domesticated animals. Commonly used approaches to diagnose babesiosis include microscopic examination of peripheral blood smears, detection of circulating antibodies and PCR. To screen and differentiate canine *Babesia* infections many PCR assays amplify the 18S rRNA gene. These sequences contain hypervariable regions flanked by highly conserved regions allowing for amplification of a broad-range of *Babesia* spp. However, differences in the 18S rRNA gene sequence of distantly related clades can make it difficult to design assays that will amplify all *Babesia* species while excluding the amplification of other eukaryotes. By targeting *Babesia* mitochondrial genome (mtDNA), we designed a novel three primer qPCR with greater sensitivity and broader screening capabilities to diagnose and differentiate *Babesia* spp.

**Methods:**

Using 13 *Babesia* mtDNA sequences, a region spanning two large subunit rRNA gene fragments (*lsu5*-*lsu4*) was aligned to design three primers for use in a qPCR assay (LSU qPCR) capable of amplifying a wide range of *Babesia* spp. Plasmid clones were generated and used as standards to determine efficiency, linear dynamic range and analytical sensitivity. Animals naturally infected with vector-borne pathogens were tested retrospectively and prospectively to determine relative clinical sensitivity and specificity by comparing the LSU qPCR to an established 18S rDNA qPCR.

**Results:**

The LSU qPCR efficiencies ranged between 92 and 100% with the limit of detection at five copies/reaction. The assay did not amplify mammalian host or other vector-borne pathogen gDNA except *Cytauxzoon felis* (a feline protozoal pathogen). The LSU qPCR assay amplified 12 different *Babesia*. sp. and *C. felis* from 31/31 (100%) archived samples, whereas the 18S qPCR amplified only 26/31 (83.9%). By prospective analysis, 19/394 diagnostic accessions (4.8%) were LSU qPCR positive, compared to 11/394 (2.8%) 18S rDNA qPCR positive.

**Conclusions:**

We have developed a more sensitive qPCR assay with a more expansive range of *Babesia* spp. detection by targeting a highly conserved region of mtDNA, when compared to an established 18S qPCR.

## Background

Babesiosis is a protozoal, tick transmitted disease found worldwide in humans, wildlife and domesticated animals. Dogs can be infected with a wide range of *Babesia* spp., including *B. gibsoni*, *B. vogeli*, *B. canis*, *B. rossi*, *B. conradae*, *B. microti*-like (also referred to as "Theileria annae" or "B. vulpes") and several large un-named *Babesia* spp., designated “*B. coco*” [1–6]. Clinical signs of canine babesiosis include thrombocytopenia, anemia, splenomegaly, fever, and can result in death [7]. Methods of diagnosing *Babesia* infections include microscopic examination of peripheral blood smears, indirect immunofluorescent antibody test to detect circulating antibodies and polymerase chain reaction (PCR) to detect pathogen DNA. Gene targets that have been used to amplify *Babesia* DNA include 18S ribosomal RNA, beta-tubulin, heat shock protein 70 (*hsp70*), thrombospondin related adhesive protein gene (P18 or BgTRAP) and two internal transcribed spacers (ITS1 and ITS2) [8–14]. Amplifying evolutionarily conserved genes, essential for survival, are often reliable PCR targets for genus-specific screening assays; however, identifying highly conserved regions for primer annealing in close proximity to regions of sequence heterogeneity for species discrimination can be challenging, particularly when including more distant lineages of *Babesia* spp. To screen and differentiate canine *Babesia* infections, the Vector-Borne Disease Diagnostic Laboratory at North Carolina State University (VBDDL-NCSU) has utilized a quantitative real-time PCR (qPCR) assay designed to amplify a region of the evolutionarily conserved 18S rRNA gene [15]. Ribosomal DNA sequences contain hypervariable regions, which are frequently used for species-specific amplification and are flanked by highly conserved regions used for broad-range genus amplification. This effectively allows amplification and discrimination of most *Babesia* species. However, differences in the 18S rRNA gene sequence of the more distantly related clades, which include *B. conradae* and *B. microti*-like parasites, make it difficult to design 18S rDNA assays that will amplify all *Babesia* species while excluding the amplification of other eukaryotes. Therefore, diagnostic laboratories often design separate and specific PCR assays to amplify *B. conradae* and *B. microti*-like in dogs where these pathogens are suspected. Screening for a wide range of *Babesia* species using specific primers for each species creates challenges for high throughput testing and limits the ability of a laboratory to identify “new” species that might infect dogs or other domestic and wild animals. In this study, we set out to design a novel assay with greater sensitivity and broader screening capabilities while retaining the ability to differentiate *Babesia* spp. This goal was achieved by targeting the *Babesia* mitochondrial genome (mtDNA).

Like other Apicomplexa*, Babesia* mtDNA can be present in higher copy numbers than the chromosomal genome and contains evolutionarily conserved genes including cytochrome b (*cytb)*, cyclooxygenase (*cox)* and large subunit ribosomal DNA (*lsu)* [16]. Improved sensitivity over the 18S rDNA target has been demonstrated using mtDNA targets in several Apicomplexa PCR assays, including *Babesia* and *Theileria* spp. [17–22]. To the authors’ knowledge this report describes the first single qPCR targeting *Babesia* mtDNA that amplifies a wide range of *Babesia* spp. We describe the development and validation of a *Babesia* genus-specific, three primer qPCR assay targeting the *lsu5*-*lsu4* region of mtDNA. The diagnostic utility of this assay (LSU qPCR) was demonstrated through retrospective and prospective analysis by comparing the sensitivity and specificity to an established 18S rDNA *Babesia* genus-specific qPCR using blood samples from uninfected and naturally-infected animals.

## Methods

### Samples

Samples of ethylenediamine tetraacetic acid (EDTA)-anticoagulated whole blood specimens from various host animals submitted to the VBDDL-NCSU for research or diagnostic testing were used to test the sensitivity and specificity of this assay. Retrospective testing was performed on archived feline, bovine, canine, equine, and wildlife samples previously characterized as containing *Babesia* spp. (*n* = 31) to assess sensitivity, and samples containing a different vector-borne pathogen (*n* = 13) or samples from uninfected animals (*n* = 4) were used to assess specificity. Archived DNA samples were previously characterized as uninfected or infected using species-specific PCRs or PCR amplification and sequence analysis of the V4 hypervariable region of the *Babesia* 18S rRNA gene [8]. Six of the characterized samples were from previously published studies and included a *B. rossi* sample, three *B. conradae* samples and two *B. microti*-like samples from grey and red foxes [23–25]. Samples containing non-*Babesia* vector-borne pathogens were confirmed by PCR amplification and sequencing by the VBDDL-NCSU using species-specific gene targets and included *Anaplasma platys*, *A. phagocytophilum*, *Bartonella henselae*, *Cytauxzoon felis*, *Ehrlichia canis*, *E. ewingii*, *Hepatozoon americanum*, *H. canis*, *Leishmania infantum*, *Mycoplasma hemocanis, Neorickettsia risticii*, *Rickettsia rickettsii*, *Theileria equi* and *Trypanosoma cruzi*. Prospective testing was performed on canine samples submitted between July 1, 2015 and August 28, 2015 for vector-borne disease testing (*n* = 394).

### Primers

**Fig. 1:**
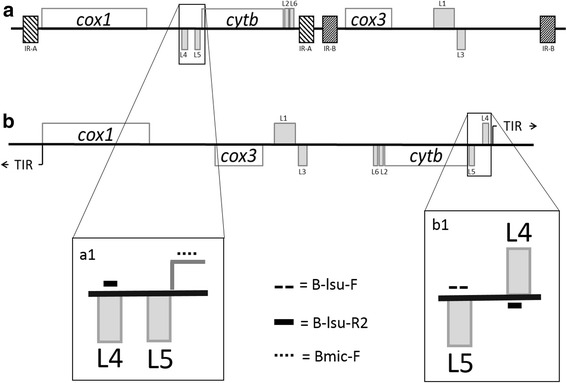
*Babesia* LSU qPCR primers in relation to the mitochondrial DNA genome structure. **a** *a1 Babesia microti* (Type-I and Type II orientation) [27] and *B. microti*-like [28]; **b** *b1* “typical” Piroplasmida [26]

**Fig. 2:**
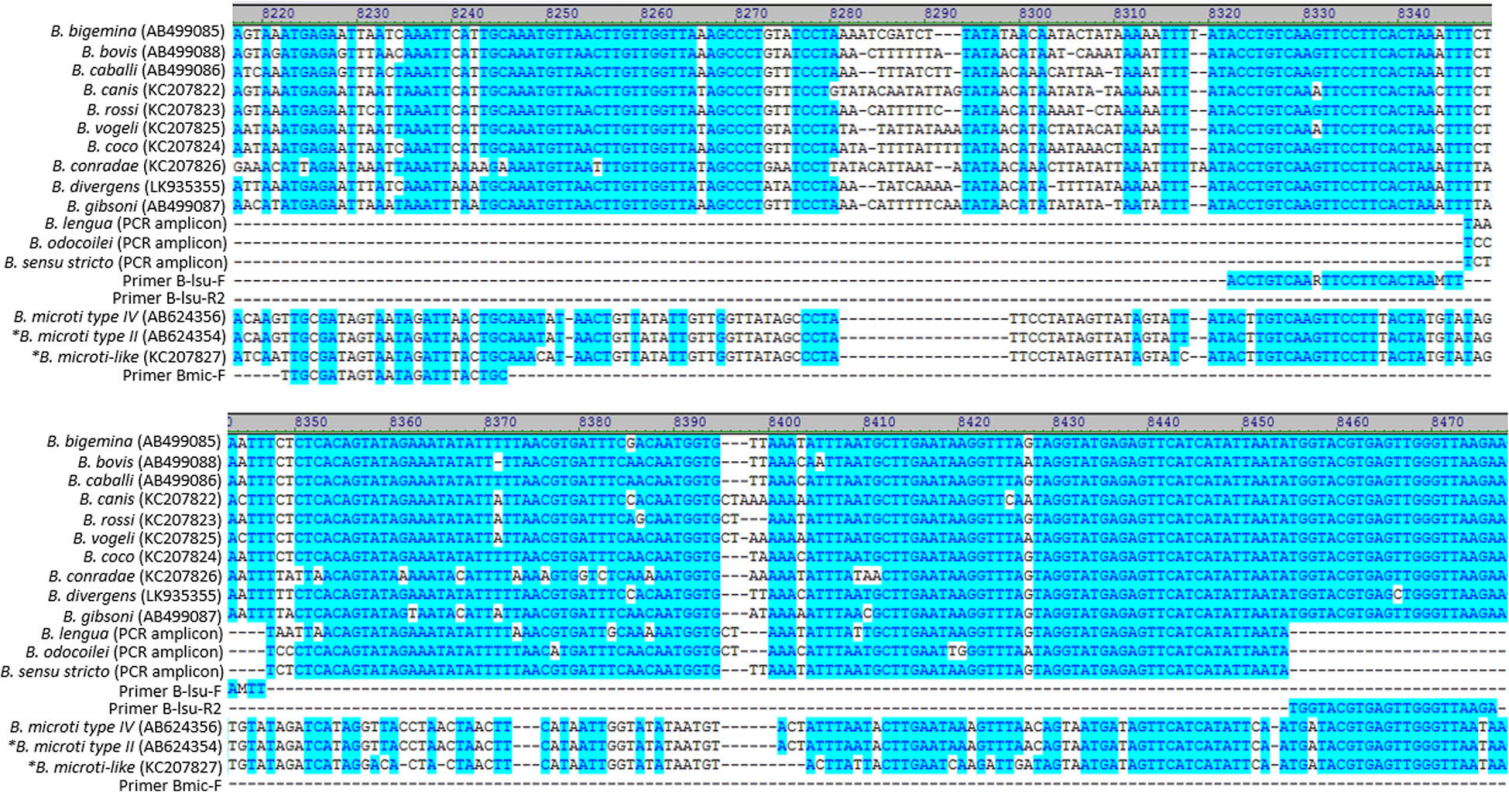
Sequence alignment of the *lsu5-lsu4* region of the mitochondrial DNA from representative *Babesia* spp. and primers designed for use in the *Babesia* LSU qPCR assay. The source of sequence for *Babesia* spp. are shown in parentheses (GenBank accession numbers or PCR amplicon sequence). * represents sequences aligned in a reverse complement orientation

To design primers better able to detect DNA from known and emerging *Babesia* spp., alignments were made between a wide range of *Babesia* mtDNA sequences. A region spanning two large subunit rRNA gene fragments (*lsu5* and *lsu4*) conserved among *Babesia* spp. containing sequence heterogeneity, flanked by areas of high similarity, was identified as a potential new qPCR target (Fig. 1) [26–28]. To develop a new *Babesia* LSU qPCR (LSU qPCR), three primers (2 forward, 1 reverse; Table 1) were designed using an alignment of the following 13 *Babesia* mtDNA sequences: *B. bovis* (AB499088), *B. bigemina* (AB499085), *B. caballi* (AB499086), *B. coco* (KC207824), *B. canis* (KC207822), *B. rossi* (KC207823), *B. vogeli* (KC207825), *B. conradae* (KC207826), *B. divergens* (LK935355), *B. gibsoni* (AB499087), *B. microti-*type II (AB624354), *B. microti*-type IV (AB624356) and *B. microti*-like (KC207827) (Fig. 2). B-lsu-F and B-lsu-R2 were designed to amplify a ~150 bp product from all *Babesia* spp. in the alignment except *B. microti* or *B. microti*-like. Bmic-F was designed to amplify a ~230 bp product from *B. microti* or *B. microti*-like when used with B-lsu-R2 (Figs. 1, 2). For assay comparisons, an established *Babesia* genus 18S rDNA qPCR (18S qPCR), utilized by the VBDDL-NCSU for *Babesia* molecular diagnostic detection, was used to amplify a ~200 bp region of the *Babesia* 18S rRNA gene with Bcommon_F and Bcommon_R primers (Table 1). Established *Babesia* species-specific PCRs using primers designed to anneal to a hypervariable region of the 18S rRNA gene and newly designed species-specific *cox*1 (a gene found on the mtDNA) primers were used to confirm *Babesia* species in LSU qPCR and 18S qPCR positive samples (Table 1). Initial amplicons generated in both the LSU qPCR and *cox*1 species-specific qPCRs from samples containing known *Babesia* spp. were sequenced to confirm primers amplified the correct target DNA for each *Babesia* species. Amplicons generated during prospective testing from the LSU-qPCR but not the 18S qPCR assay (discordant results) were sequenced.

**Table 1:** Primer sequences for *Babesia* genus and species-specific PCRs

Primer name	Gene target	Sequence (5′-3′)
B-lsu-F	*Babesia lsu5-lsu4*	ACCTGTCAARTTCCTTCACTAAMTT
B-lsu-R2	*Babesia lsu5-lsu4*	TCTTAACCCAACTCACGTACCA
Bmic-F	*B. microti-*like *lsu5-lsu4*	TTGCGATAGTAATAGATTTACTGC
Bcommon-F	*Babesia* 18S rRNA	GCATTTGCGATGGACCATTCAAG
Bcommon-R	*Babesia* 18S rRNA	CCTGTATTGTTATTTCTTGTCACTACCTC
BMIC18-F	*B. microti-*like 18S rRNA	CTGCTTTATCATTAATTTCGCTTCCGAACG
BCV-F	*B. vogeli* 18S rRNA	GTTCGAGTTTGCCATTCGTT
BCC-F	*B. canis* 18S rRNA	TTGCGTTGACGGTTTGACC
BCO-F	*B. coco* 18S rRNA	CCTTTTCTTTGCTTTGTCGC
BGNC-F	*B. gibsoni* 18S rRNA	ACTCGGCTACTTGCCTTGTC
BAB722-R	*Babesia* 18S rRNA	ATGCCCCCAACCGTTCCTATTA
BCV-cox1-F4	*B. vogeli cox*1	TGCTATGAGTGGCGCAAATTTTG
BCV-cox1-R	*B. vogeli cox*1	CCATACAGTAGGTATCAATCTATCT
BCC-cox1-F2	*B. canis cox*1	GTGCAATGAGTGGAGCAAATTTCA
BCC-cox1-R	*B. canis cox*1	CCATACAGTTGGTATTAATCTATCC
BCO-cox1-F2	*B. coco cox*1	TTGTAACTTCTGTTTTACTTATGGTG
BCO-cox1-R2	*B. coco cox*1	AAAATAAGAATATAAACCTCAGGATGT
BG-cox1-F	*B. gibsoni cox*1	CTTCAGCCAATAGCTTTCTGTTTG
BG-cox1-R	*B. gibsoni cox*1	CCTGAGGCAAGTAAACCAAATAT

### DNA extraction and PCR conditions

DNA extraction was performed on QIAsymphony^SP^ (Qiagen, Hilden, Germany) with QIAsymphony® DNA Mini Kit (192) (Qiagen) or Qiagen BioRobot® M48 Robotic Workstation with MagAttract® DNA Mini M48 kit (Qiagen) depending on the time of sample submission. DNA was stored at -20 °C until PCR analysis. All PCR sample preparations were prepared in a biocontainment hood with UV light decontamination capabilities. The absence of PCR inhibitors was demonstrated by the amplification of GAPDH (glyceraldehyde-3-phosphate dehydrogenase) [8]. Amplification reactions for all PCRs contained 12.5 μl SYBR®Green Supermix (Bio-Rad, Hercules, USA), 5 μl DNA template, primers at various concentrations (Table 2) and molecular grade water to a final volume of 25 μl. Thermocycler conditions were established based on several factors, which included using temperature cycles that could be run with other NCSU-VBDDL qPCR assays, recommendations by Bio-Rad for use of SYBR®Green Supermix, and calculated melting temperatures of newly designed primers. Furthermore, gradients for primer annealing temperatures and concentrations were performed to identify a combination that amplified target DNA efficiently but did not amplify nonspecific DNA. Thermocycler conditions consisted of an initial denaturation step at 98 °C for 3 min, followed by 40 cycles at 98 °C for 15 s, 60 °C for 15 s (or 62 °C for *B. microti*-like 18S species-specific qPCR), and 72 °C for 15 s. Melting temperature (*T*_*m*_) measurements were made between 65 and 88 °C at 0.5 s intervals. All qPCRs included a positive control consisting of either a previously characterized *Babesia*-infected sample or *Babesia* plasmid DNA and negative controls including a no-template control consisting of filter-sterilized, molecular-grade water and uninfected dog or cat genomic DNA (gDNA). Newly extracted samples were tested with DNA extraction controls. PCR amplification was performed in a C1000™ Thermal Cycler (Bio-Rad) with CFX96™ Real-Time Detection System. Following PCR, amplicons were analyzed via the quantification cycle (C_q_), melt curve shape and *T*_*m*_ difference. To validate amplicon size, several PCR products were visualized on a 2.0% agarose gel in 1× TAE with ethidium bromide staining alongside a DNA molecular size marker. Sequencing of products or plasmids was performed by GENEWIZ Inc. (Research Triangle Park, NC) and alignments made with GenBank reference sequences using AlignX software (Vector NTI Suite 6.0, InforMax, Inc.)

**Table 2:** Primer combinations and concentrations used for species of the genus *Babesia* and species-specific (sp-sp) PCRs

qPCR	Primer combination (μM)
*Babesia* genus LSU qPCR	B-lsu-F (0.6); B-lsu-R2 (0.6); Bmic-F (0.4)
*Babesia* genus 18S qPCR	Bcommon-F (0.4); Bcommon-R (0.4)
*B. microti*-like sp-sp 18S qPCR	BMIC18-F (0.8); BAB722 (0.8)
*B. vogeli* sp-sp 18S qPCR	BCV-F (0.4); BAB722 (0.4)
*B. canis* sp-sp 18S qPCR	BCC-F (0.4); BAB722 (0.4)
*B. coco* sp-sp 18S qPCR	BCO-F (0.4); BAB722 (0.4)
*B. gibsoni* sp-sp 18S qPCR	BGNC-F (0.4); BAB722 (0.4)
*B. vogeli* sp-sp *cox*1 qPCR	BCV-cox1-F4 (0.4); BCV-cox1-R (0.4)
*B. canis* sp-sp *cox*1 qPCR	BCC-cox1-F2 (0.4); BCC-cox1-R (0.4)
*B. coco* sp-sp *cox*1 qPCR	BCO-cox1-F2 (0.4); BCO-cox1-R2 (0.4)
*B. gibsoni* sp-sp *cox*1 qPCR	BG-cox1-F (0.4); BG-cox1-R (0.4)

### qPCR efficiency

Plasmid clones, used as standards for efficiency and analytical sensitivity determination, were constructed using LSU qPCR amplicons from *B. vogeli*, and *B. microti*-like template DNA with pGEM-T Easy Vector System (Promega, Madison, WI) as recommended by the manufacturer. Plasmids were sequenced and inserts confirmed using M13R primers. Plasmid copy numbers were calculated assuming an average base pair weight of 650 Da and Avogadro’s number (6.022 10^23^) using the following equation: copy number = (DNA ng amount × 6.022 10^23^ molecules/mol)/(length of DNA in base pairs × 1 × 10^9^ ng/g × 650 g/mol). Duplicate, serial 10-fold dilutions in uninfected canine gDNA (~10–30 ng/μl) resulted in 50–500,000 copies/reaction of plasmid DNA, and standard curves of quantification cycle (C_q_) values were plotted against the logarithm of plasmid copy numbers/reaction. PCR efficiency was estimated through linear regression of the dilution curve (10^ (-1/slope)-1) × 100). Coefficients were calculated (*R* ^2^) using Bio-Rad CFX Manager™ software. Efficiency reactions were performed using both 2 and 3 primer reactions to establish any potential interference by a third primer.

### Analytical sensitivity and specificity

Analytical sensitivity was determined by calculating the limit of detection (LOD), defined as the lowest concentration at which 95% of the positive samples were detected. Plasmids diluted in canine gDNA (~10–30 ng/μl) to 1 copy/μl were added to reaction wells resulting in 3 and 5 copies/reaction. Twenty intra-assay replicates for each plasmid concentration were tested and C_q_ ranges determined. All assays were performed using both 2 and 3 primer reactions to establish any potential interference by a third primer. Analytical specificity was evaluated retrospectively using gDNA (~10–30 ng/μl) previously extracted from bovine, canine, equine and feline EDTA-whole blood specimens and tested by the VBDDL, determined to be either uninfected or infected with non-*Babesia* vector-borne pathogens.

### Clinical sensitivity and specificity

To determine relative clinical sensitivity and specificity, the LSU qPCR was compared to an established *Babesia* 18S qPCR diagnostic assay that has been used for over 12 years. Retrospective and prospective testing was performed. Retrospective analysis included 31 archived gDNA samples, previously characterized as positive with 12 different *Babesia* spp. including *B. bovis*, *B. caballi*, *B. canis*, *B. rossi*, *B. vogeli*, *B. coco*, *B. conradae*, *B. gibsoni*, *B. lengau*, *B. microti*-like, *B. odocoilei*, and two *Babesia* species in the *Babesia* (*sensu stricto*) clade, or *C. felis*. After amplification with the LSU qPCR, one of each of the 12-designated species from the retrospective sample set was sequenced and aligned with a reference sequence for species confirmation. Prospective analysis included 394 canine diagnostic specimens submitted to the VBDDL for testing using a comprehensive vector-borne disease PCR panel, or when specifically requested, *Babesia* PCR alone. All samples were simultaneously tested using the 18S qPCR assay and the LSU qPCR assay. Prospective specimens were tested at the time of submission and all PCR positive results were speciated with additional, species-specific PCRs targeting 18S rRNA or the *cox*1 gene. In addition, any discordant PCR positive amplicons from the prospective study were sequenced to confirm results and were retested in triplicate with both PCRs to account for the effect of Poisson distribution on samples with low template concentrations.

### Statistical analysis

Relative sensitivity and specificity for the 18S qPCR and LSU qPCR were determined by a 2 × 2 table, calculating an estimate of agreement relative to a non-reference standard, either the 18S qPCR or the LSU qPCR [29]. Positive percent agreement (PPA), representing relative sensitivity, was determined by the proportion of non-reference standard positive samples where the index assay is positive [a/(a + c)], and negative percent agreement (NPA), representing relative specificity, was determined by the proportion of non-reference standard negative samples where the index test is negative [d/(b + d)]. Agreements and proportions were reported with their 95% confidence intervals (95% CI), calculated by the modified Wald method [30, 31]. Confidence intervals were performed using GraphPad Software (La Jolla, California, USA).

## Results

### qPCR efficiency, analytical sensitivity and specificity

**Fig. 3:**
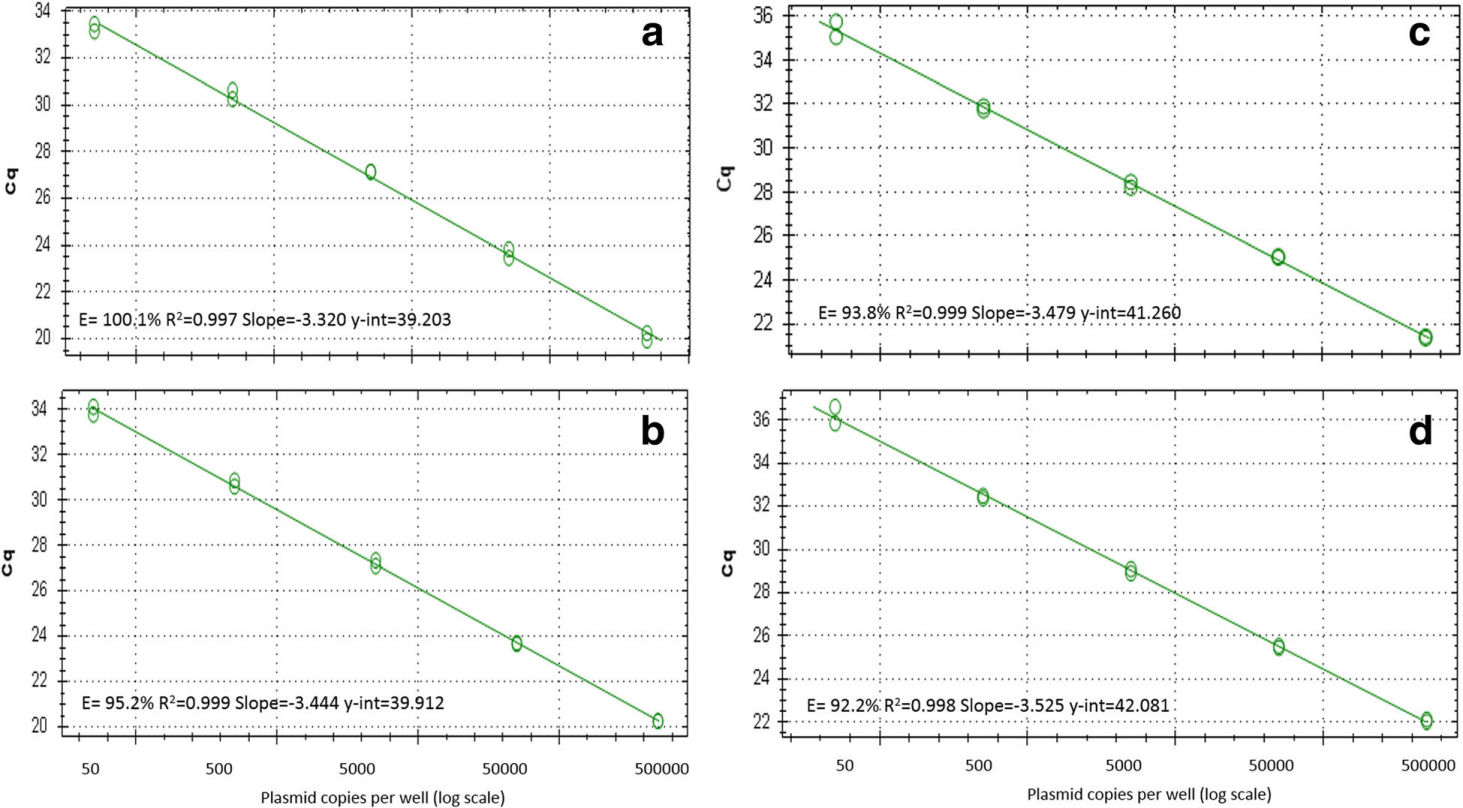
Efficiency curves for the *Babesia* LSU qPCR using plasmids as template DNA. **a** *B. microti*-like plasmid (pBMIC-LSU) with 2 primers (Bmic_F and B-lsu-R2). **b** pBMIC-LSU with 3 primers (Bmic-F, B-lsu-F and B-lsu-R2). **c** *B. vogeli* plasmid (pBCV-LSU) with 2 primers (B-lsu-F and B-lsu-R2). **d** pBCV-LSU with 3 primers (Bmic-F, B-lsu-F and B-lsu-R2). *Abbreviation*: E, efficiency

Amplicon size for each primer pair was visualized on an agarose gel and corresponded to the expected size of ~150 bp for most *Babesia* spp. and ~230 bp for *B. microti*-like. *T*_*m*_ ranged from 76 to 77.5. LSU qPCR efficiencies ranged from 92 to 100% with an R^2^ of 0.99 and a linear dynamic range of 50–500,000 copies/reaction of plasmid DNA (Table 3, Fig. 3). Results were comparable when using either two primers (Bmic-F with B-lsu-R2 for *B. microti*-like plasmid template or B-lsu-F with B-lsu-R2 for *B. vogeli* plasmid template) or three primers (Bmic-F, B-lsu-F and B-lsu-R2) (Table 3). LODs were initially tested at 3 copies/well using 20 intra-assay replicates, with detection ranging from 60 to 90% (Table 3). When the LOD was repeated at 5 copies/well, all reactions showed 100% detection except the 3 primer *B. microti*-like reaction (95% detection). Neither the LSU qPCR nor the 18S qPCR amplified uninfected mammalian host DNA. The LSU qPCR did not amplify non-*Babesia* vector-borne pathogens except for *C. felis*, and the 18S qPCR amplified *C. felis*, *H. canis* and *T. equi* (Table 4).

**Table 3:** The efficiency and analytical sensitivity for the *Babesia* LSU qPCR. The efficiency and analytical sensitivity were determined for the *Babesia* LSU qPCR using plasmids as template DNA run in 20 intra-assay replicates at 3 copies/well and 5 copies/well. Assays were run with all 3 primers (Bmic-F, B-lsu-F and B-lsu-R2) for both plasmids and each corresponding 2 primer reaction: Bmic-F with B-lsu-R2 for *B. microti*-like plasmid (pBMIC-LSU) and B-lsu-F with B-lsu-R2 for *B. vogeli* plasmid (pBCV-LSU)

Primers	Plasmid template	Eff (%)	*R* ^2^	3 c/well (%)	Cq (range)	5 c/well (%)	Cq (range)
2 primers	pBMIC-LSU	100	0.99	90	34.1–37.2	100	33.5–37.5
3 primers	pBMIC-LSU	95	0.99	70	33.6–37.3	95	34.2–37.8
2 primers	pBCV-LSU	94	0.99	60	37.1–39.9	100	33.3–36.7
3 primers	pBCV-LSU	92	0.99	60	36.8–39.2	100	33–34.2

*Abbreviations:* *Eff* efficiency, *C* copies

**Table 4:** Retrospective analysis qPCR results. Retrospective qPCRs were performed simultaneously on previously characterized diagnostic or research samples from mammals that were uninfected (*n* = 4), naturally infected with *Babesia* or *Cytauxzoon* species (*n* = 31) or other vector-borne pathogens (*n* = 13)

Characterized sample (source)	*Babesia* 18S qPCR		*Babesia* LSU qPCR	
	C_q_	T_*m*_ (°C)	C_q_	T_*m*_ (°C)
*B. bovis* (cow)	32.2	86.5	17.0	76.5
*B. caballi* (horse)	29.4	86	29	77
*B. caballi* (horse)	36.3	86	35.1	76.5
*B. canis* (dog)	33.7	86	32.9	77
*B. canis* (dog)	36.7	86	36.8	77
*B. canis* (dog)	32.5	86	31.4	77
*B. rossi* (dog)	23.7	86	22.8	76.5
*B. vogeli* (dog)	21.9	86.5	20.5	77
*B. vogeli* (dog)	21.1	86	17.8	76.5
*B. coco* (dog)	34.7	86	27.8	77
*B. coco* (dog)	32.1	86	25.1	77
*B. coco* (dog)	–	none	40.6	77
*B. conradae* (dog)	34.2	86	19.8	76
*B. conradae* (dog)	39	86	19.7	76
*B. conradae* (dog)	–	none	17.3	76
*B. conradae* (dog)	–	none	21.4	76.5
*B. gibsoni* (dog)	13.2	86	13.01	77
*B. gibsoni* (dog)	19.3	86	19.2	77
*B. lengau* (leopard)	38.2	85.5	18	76
*B. microti*-like (dog)	–	none	30.8	77
*B. microti*-like (dog)	–	none	40.3	76.5
*B. microti*-like (dog)	32	86	24	76.5
*B. microti*-like (red fox)	36.5	85.5	27.8	76
*B. microti*-like (grey fox)	36.6	85.5	28.2	76
*B. odocoilei* (reindeer)	16.5	86	23.9	77
*B. odocoilei* (reindeer)	13.5	86.5	25.5	77
*B. odocoilei* (elk)	24.9	86.5	35.6	76.5
*B.* (*sensu stricto*) (maned wolf)	14.4	86	13.6	77
*B.* (*sensu stricto*) (bear)	34.1	86	32.4	76.5
*Cytauxzoon felis* (cat)	31.8	85.5	17.2	77.5
*C. felis* (cat)	35.3	85.5	21.2	77.5
Uninfected gDNA (cat)	–	none	–	none
Uninfected gDNA (cow)	–	none	–	none
Uninfected gDNA (dog)	–	none	–	none
Uninfected gDNA (horse)	–	none	–	none
*Anaplasma phagocytophilum* (dog)	–	none	–	none
*A. platys* (dog)	–	none	–	none
*Bartonella henselae* (cat)	–	none	–	none
*Ehrlichia canis* (dog)	–	none	–	none
*E. ewingii* (dog)	–	none	–	none
*Hepatozoon canis* (dog)	39.0	85	–	none
*H. americanum* (dog)	–	none	–	none
*Leishmania infantum* (dog)	–	none	–	none
*Mycoplasma hemocanis* (dog)	–	none	–	none
*Neorickettsia risticii* (culture)	–	none	–	none
*Rickettsia rickettsii* (dog)	–	none	–	none
*Theileria equi* (horse)	38.9	86	–	none
*Trypanosoma cruzi* (dog)	–	none	–	none

*Abbreviations:* *C*_*q*_ quantification cycle, *T*_*m*_ melting temperature; −, *Babesia* was not amplified

### Clinical sensitivity and specificity

**Fig. 4:**
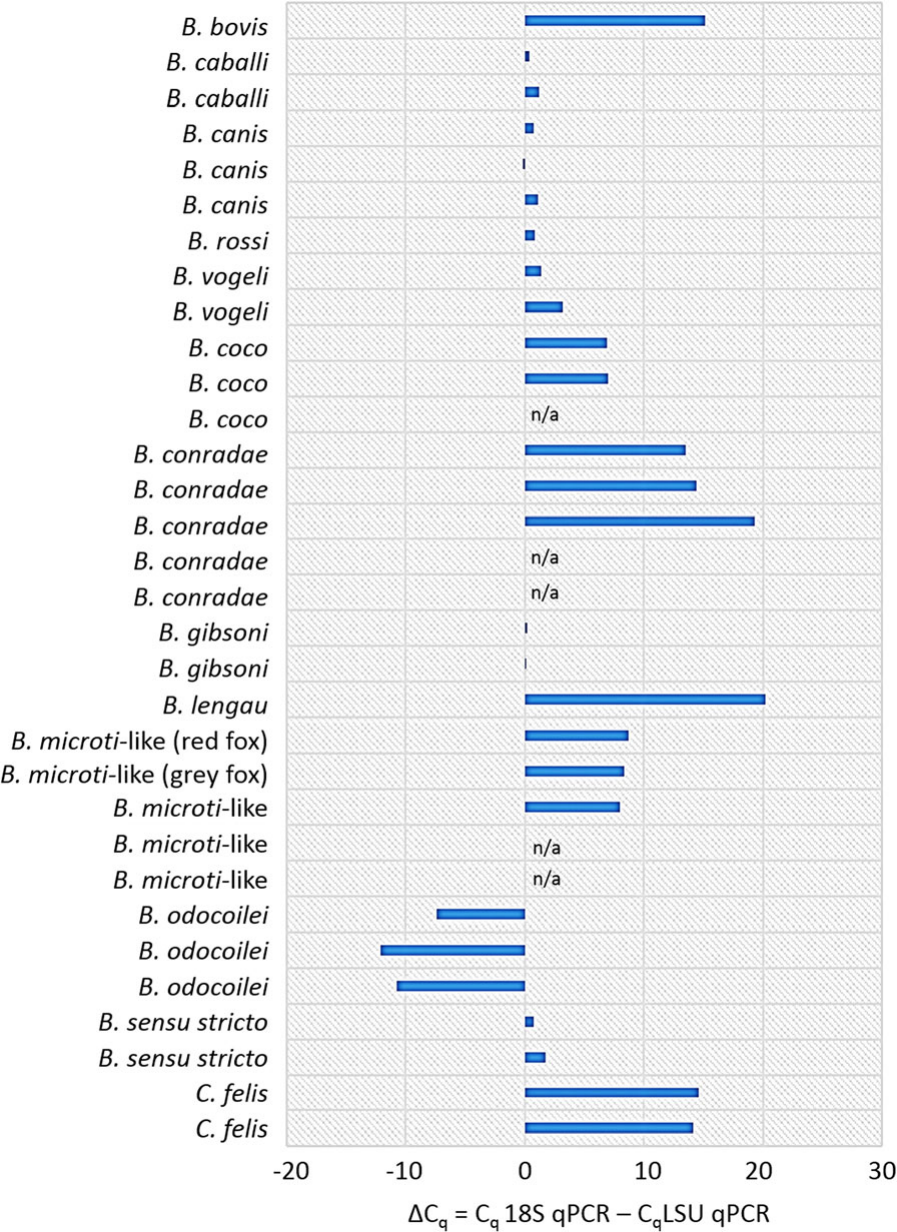
Comparison of the ΔC_q_ from retrospective 18S qPCR and LSU qPCR assays performed simultaneously on previously characterized diagnostic or research samples naturally infected with *Babesia* spp. and *C. felis*. n/a = sample was negative by 18S qPCR and positive in LSU qPCR; ΔC_q_ = C_q_ 18S qPCR – C_q_ LSU qPCR

Of the 31 archived, naturally-infected animal blood samples, previously characterized as containing 12 different *Babesia* spp. or *C. felis,* 31/31 (100%) were LSU qPCR positive and 26/31 (83.9%) were 18S qPCR positive (Table 4). The sequenced LSU qPCR amplicons from 9/12 *Babesia* species and *C. felis* were 100% identical with GenBank reference sequences. *Babesia lengau*, *B. odocoilei* and *Babesia* (*sensu stricto*) mtDNA reference sequences were not available in GenBank for comparison and were aligned with the *lsu5-lsu4* region from other Babesia mtDNA sequences (Fig. 2). Of the 26 concordant samples, the average C_q_ value difference between the two qPCRs (ΔC_q _ = 18S qPCR C_q_ – LSU qPCR C_q_) was 7.1. The LSU qPCR C_q_ was less than the 18S qPCR C_q_ (representing earlier amplicon production) for *B. bovis* (ΔC_q_ = 15.2), *B. coco* (ΔC_q_ = 6.9 and 7.0), *B. conradae* (ΔC_q_ = 13.5, 14.5, and 19.3), *B. microti*-like (ΔC_q_ = 8.0, 8.4, and 8.7), *B. lengau* (ΔC_q_ = 20.2) and *C. felis* (ΔC_q_ = 14.2 and 14.6) (Fig. 4). *Babesia odocoilei* was the only *Babesia* species in this sample set where the 18S qPCR amplified DNA at a lower C_q_ (ΔC_q_ = -10.7, -12.1 and -7.35) than the LSU qPCR.

Of the 394 canine diagnostic specimens submitted for vector-borne disease testing, 2.8% (11/394; 95% CI: 1.1–4.4%) were *Babesia*-positive by the 18S qPCR and 4.8% (19/394; 95% CI: 3.1–7.5%) were positive by the LSU qPCR (Table 5). All but one of the positive samples were speciated using species-specific PCRs targeting the 18S rRNA or *cox*1 gene (Table 6). The *Babesia* spp. that tested positive by both qPCR assays included *B. canis* (*n* = 2), *B. vogeli* (*n* = 2) and *B. gibsoni* (*n* = 7) (Table 6). Discordant results were obtained for 8 samples that were 18S qPCR negative, but LSU qPCR positive, including *B. coco* (*n* = 2), *B. gibsoni* (*n* = 5) and *B. microti*-like (*n* = 1) (Table 6). All but one of the discordant samples were confirmed by *cox*1 species-specific qPCRs and amplicon sequencing of the LSU qPCR product. Two of the discordant samples were also positive by 18S species-specific qPCRs (Table 6). Of the discordant samples that were retested in triplicate with the LSU qPCR, 4 were positive in 3/3 replicates, 2 were positive in 2/3 replicates, 1 was positive in 1/3 replicates and 1 was negative in 3/3 replicates. For the 18S qPCR triplicate retest, 7 discordant samples were negative again in 3/3 replicates and 1 (*B. gibsoni*) was positive in 1/3 replicates (Table 6).

**Table 5:** Positive LSU qPCR results were compared with 18S qPCR results from a prospective analysis performed on canine diagnostic samples naturally infected with *Babesia* spp.

Total samples (*n* = 394)	18S	LSU
*B. canis* (+)	2	2
*B. vogeli* (+)	2	2
*B. coco* (+)	0	2
*B. gibsoni* (+)	7	12
*B. microti*-like (+)	0	1
% Positive (95% CI)	2.8 (1.1–4.4%)	4.8 (3.1–7.5%)

(+) = C_q_ value obtained, *T*_*m*_ value was correct

**Table 6:** Prospective analysis was performed on canine diagnostic samples naturally infected with *Babesia* spp. and quantification cycles (C_q_) were compared between the 19 positive samples detected by *Babesia* genus assays (18S qPCR or LSU qPCR) and species-specific assays (18S sp-sp or *cox*1 sp-sp). Results are shown from the original Babesia genus qPCRs, discordant samples that were repeated in triplicate with the *Babesia* genus qPCRs, and *Babesia* species-specific qPCRs. All positive samples generated the correct melting temperature (*T*_*m*_) values (not shown)

	Genus level qPCR				Species-specific qPCR	
	Original results		Repeated results (triplicate)			
*Babesia* spp.	18S	LSU	18S	LSU	18S	*cox*1
*B. canis*	31.4	30.5	na	na	31.6	30.0
*B. canis*	31.7	29.9	na	na	31.8	31.3
*B. vogeli*	30.6	29.4	na	na	31.3	31.4
*B. vogeli*	32.1	32.1	na	na	31.1	31.7
*B. gibsoni*	13.8	13.9	na	na	14.8	16.3
*B. gibsoni*	17.7	19.6	na	na	19.5	22.9
*B. gibsoni*	19.6	18.0	na	na	21.8	24.4
*B. gibsoni*	21.6	22.2	na	na	30.1	23.0
*B. gibsoni*	29.2	30.4	na	na	29.2	30.8
*B. gibsoni*	18.0	18.0	na	na	19.3	19.7
*B. gibsoni*	13.5	13.3	na	na	14.5	15.8
*B. coco*	–	39.8	–	38.5; 38.6; 33.8	–	39.0
*B. coco*	–	38.2	–	39.2; 33.1; −	–	36.0
*B. gibsoni*	–	37.5	–	–	–	–
*B. gibsoni*	–	39.4	–	40.7; 38.3; 38.4	39	37.0
*B. gibsoni*	–	30.9	–	29.4; 29.7; 30.6	–	31.1
*B. gibsoni*	–	39.0	37.0; −; −	38.4; −; −	–	36.2
*B. gibsoni*	–	38.4	–	39.5; 38.6; −	–	39.0
*B. microti-like*	–	29.0	–	31.0; 30.6; 30.3	32	na

*Abbreviations:* na, the sample was not retested; −, *Babesia* was not amplified

Relative sensitivity and specificity between the 18S qPCR and LSU qPCR were determined by calculating the PPA and NPA for each assay [29] (Table 7). When using the 18S qPCR as a non-reference standard, the relative sensitivity (PPA) and specificity (NPA) of the LSU qPCR was 100% (95% CI: 69.9–100%) and 98% (95% CI: 95.9–99.0%), respectively. When using the LSU qPCR as a non-reference standard, the relative sensitivity (PPA) and specificity (NPA) for the 18S qPCR was 58% (95% CI: 36.2–76.9%) and 100% (95% CI: 98.8–100%), respectively.

**Table 7:** Positive percent agreement (PPA) and negative percent agreement (NPA)

	Positive	Negative	Row sum
LSU qPCR (index)	18S qPCR (non-reference standard)		
Positive^a^	11	8	19
Negative^b^	0	375	375
Column sum	11	383	394
18S qPCR (index)	LSU qPCR (non-reference standard)		
Positive^c^	11	0	11
Negative^d^	8	375	383
Column sum	19	375	394

*Note*: We calculated PPA and NPA using 18S qPCR assay (top) or LSU qPCR assay as the non-reference standard

^a^PPA = 100% (95% CI: 69.9–100%)

^b^NPA = 98% (95% CI: 95.9–99.0%)

^c^PPA = 58% (95% CI: 36.2–76.9%)

^d^NPA = 100% (95% CI: 98.8–100%)

## Discussion

In this report, we describe the development and validation of a sensitive and specific broad-range molecular diagnostic assay for detecting *Babesia* infections using a three-primer qPCR assay targeting the mtDNA. Prior to validation of the LSU qPCR, the VBDDL utilized a qPCR targeting the 18S rRNA gene, optimized to detect *Babesia* species infective to dogs, which included *B. canis*, *B. gibsoni*, *B. rossi* and *B. vogeli* [15]. Since development of the 18S qPCR assay, emerging *Babesia* species have been discovered to infect dogs, including *B. conradae*, *B. microti*-like and several large un-named *Babesia* spp., designated *B. coco* [5–7]. Furthermore, diagnostic laboratories, including NCSU-VBDDL, routinely test for emerging *Babesia* spp. in samples collected from other animals, such as wildlife. At high pathogen DNA quantities, the 18S qPCR can amplify 18S ribosomal DNA from these emerging species; however, amplification efficiency is poor with lower pathogen loads. Other *Babesia* diagnostic methods include testing with multiple, individual, species-specific PCR assays, reverse line blotting, or nested PCR-restriction fragment length polymorphism analysis. These approaches are either costly, not as conducive to a high throughput platform, or may not detect novel *Babesia* pathogens [32, 33]. Screening diagnostic samples at the genus level using broad-range primers followed by additional analysis of PCR positive samples, such as species-specific PCRs and/or amplicon sequencing to determine the species, supports a high throughput platform, is cost effective, and facilitates the discovery of “new” pathogens. Identifying a DNA target conserved among *Babesia* that contains hypervariable sequences flanked by highly conserved sequences, where primers do not amplify other eukaryotic DNA can be challenging. The LSU qPCR assay amplifies a region of DNA spanning two large ribosomal subunits, *lsu5*-*lsu4,* that is conserved among piroplasmida indicating it is less likely to be deleted or changed due to DNA rearrangements or mutations [26–28]. This study demonstrated an increased level of relative sensitivity and a broader range of *Babesia* spp. detection when the LSU qPCR was compared to an established molecular diagnostic assay. There was improved detection when testing animal samples naturally infected with *B. bovis*, *B. coco*, *B. conradae*, *B. lengau*, and *B. microti*-like organisms*.* While we did not include a *C. felis* mtDNA sequence (GenBank accession no. KC207821) in the *Babesia* alignment used to design new primers, the B-lsu-F and B-lsu-R2 primers are 100% identical to the corresponding sequences of the *C. felis lsu5*-*lsu4* region and amplified this feline pathogen*.* For differentiation of *C. felis* from *Babesia* spp., the authors recommend either amplicon sequencing or testing the sample with a validated *C. felis* species-specific PCR [17].

**Fig. 5:**
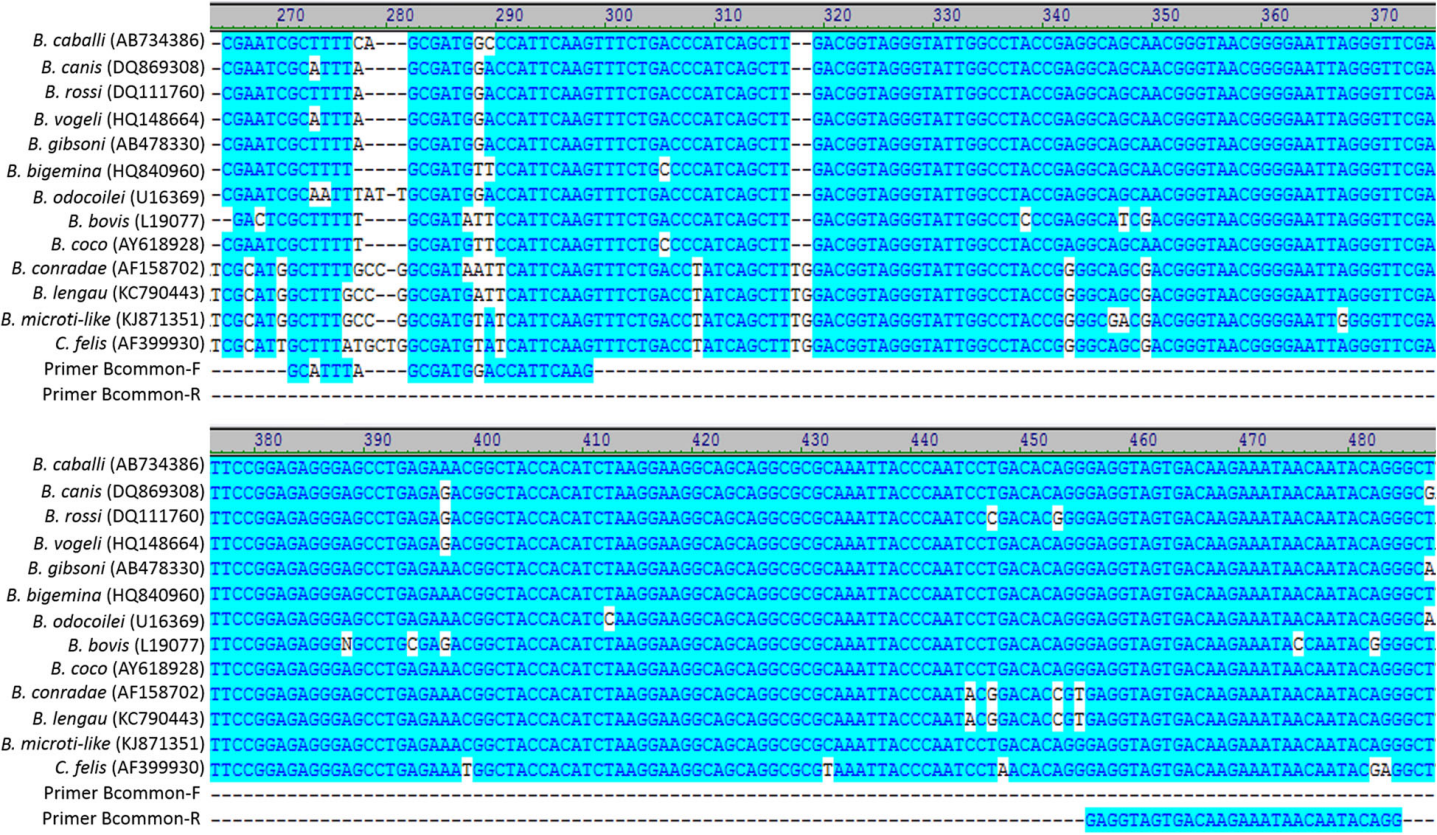
Sequence alignment of the 18S rRNA gene from representative *Babesia* spp., *Cytauxzoon felis* and primers designed for use in the *Babesia* 18S qPCR assay. The GenBank accession numbers are shown in parentheses

The C_q_ differences (ΔC_q_ = 18S qPCR C_q_ - LSU qPCR C_q_) between the 18S qPCR and the LSU qPCR have several possible explanations (Table 1, Fig. 4). It is possible the noted differences were due in part to differing primer efficiencies and not necessarily related to target DNA copy number. Primer alignments using the 18S qPCR primers, Bcommon-F (bp = 24) and Bcommon-R (bp = 29), revealed base pair mismatches were greatest with the Bcommon-F primer in *B. conradae* (9/24) *B. lengau* (8/24), *B. microti*-like (7/24), and *C. felis* (9/24), while all the other *Babesia* spp. in the retrospective sample set had bp mismatches ≤ five (Fig. 5). Except for *B. microti* and *B. microti*-like, where a different forward primer is used for the LSU qPCR target, the B-lsu-F primer has only two mismatches with *B. canis* and *B. vogeli*. However, these mismatches are mitigated with two degenerate primer nucleotides (Fig. 2). The B-lsu-R2 primer (bp = 22) has no mismatches except one in *B. divergens* and two in *B. microti* and *B. microti*-like.

A large prospective set of samples from dogs suspected of exposure to vector-borne pathogens was used to measure relative sensitivity and specificity between an established diagnostic qPCR and a newly developed qPCR. The newly developed LSU qPCR relative sensitivity was 100% when compared to the established 18S qPCR, while the 18S qPCR relative sensitivity was 58% when compared to the LSU qPCR, suggesting the new assay is more sensitive at detecting *Babesia*. The LOD for the LSU qPCR assay was determined to be ~ five copies/reaction while the established Babesia 18S rDNA was determined to be between five and ten copies/reaction (data not included); both LODs were determined using plasmids containing one copy of the target DNA diluted in canine gDNA. Making direct comparisons between LODs with other qPCRs reported in the literature using similar methods of calculation show the LSU and 18S qPCRs to be comparable. One assay targeting *B. canis hsp70* calculated a LOD at ~ ten copies using a probe for detection [12]; another qPCR assay targeting *B. microti* 18S rDNA calculated ~3.6 copies per reaction using blood spiked with plasmid DNA [34]. Both the LSU and 18S qPCR assays had high specificities relative to each other, with the 98% specificity reported for LSU qPCR reflecting the positive discordant samples not detected by the 18S qPCR.

When comparing results from naturally infected canine samples used for the prospective study, eight samples were PCR negative by the 18S qPCR but positive by the LSU qPCR (Table 7). It is unclear if this difference in sensitivity is related to improved primer binding and subsequent amplification, increased target copy number, or clinical status of the patient at time of sampling (treated or untreated). It’s possible mtDNA copy numbers vary among *Babesia* species or stages of piroplasmida infection. Schreeg et al. detected higher mtDNA copy numbers in cats acutely infected with *C. felis* when compared to cats infected over a year [17]. It remains unclear if there is a link between the mtDNA copy number and stage of *Babesia* infection.

Limitations of this study include the inability to calculate true sensitivity and specificity for the new LSU qPCR assay and that the new assay was only compared to one other qPCR assay. However, in both the prospective and retrospective sample sets, the LSU qPCR outperformed the 18S qPCR. While reasons behind this improvement remain unclear, we hypothesis that increased target copy number for the mitochondrial PCR is a contributing factor.

## Conclusions

In summary, we have developed a qPCR assay with increased sensitivity to detect a broader number of *Babesia* spp. The LSU qPCR targets a highly conserved region of the mtDNA spanning the *lsu5-lsu4* region. Retrospective and prospective analysis with samples from naturally infected animals highlight the expansive range of *Babesia* spp. detection and improved relative sensitivity when compared to a current 18S qPCR. Currently the VBDDL has implemented the new LSU qPCR assay for all diagnostic *Babesia* PCRs and confirms all positive samples with a second species-specific qPCR, targeting either the 18S rRNA or *cox*1 genes. Samples from ungulates are also screened with the 18S qPCR for improved detection of *B. odocoilei*.

## Abbreviations

18S qPCR: qPCR assay targeting a region of the 18S rRNA gene in *Babesia* spp.
C_q_: Quantification cycle
EDTA: Ethylenediamine tetraacetic acid
gDNA: Genomic DNA
LSU qPCR: qPCR assay targeting a region of the large subunit rRNA gene fragments *lsu5*- *lsu4* on the mtDNA of *Babesia* spp.
mtDNA: Mitochondrial DNA
NPA: Negative percent agreement
PPA: Positive percent agreement
qPCR: Quantitative real-time polymerase chain reaction
T_*m*_: Melting temperature
VBDDL: Vector-Borne Disease Diagnostic Laboratory

## References

[1] Patton WS (1910). Preliminary report on a new piroplasm (*Piroplasma gibsoni* sp. nov.) found in the blood of the hounds of the Madras Hunt and subsequently discovered in the blood of the jackal *Canis aureus*. Bull Soc Pathol Exot.

[2] Carret C, Walas F, Carcy B, Grande N, Précigout E, Moubri K (1999). *Babesia canis, Babesia canis vogeli, Babesia canis rossi*: differentiation of the three subspecies by a restriction fragment length polymorphism analysis on amplified small subunit ribosomal RNA genes. J Eukaryot Microbiol.

[3] Conrad PA, Thomford J, Yamane I, Whiting J, Bosma L, Uno T (1991). Hemolytic anemia caused by *Babesia gibsoni* infection in dogs. J Am Vet Med Assoc.

[4] Birkenheuer AJ, Neel J, Ruslander D, Levy MG, Breitschwerdt EB (2004). Detection and molecular characterization of a novel large *Babesia* species in a dog. Vet Parasitol.

[5] Kjemtrup AM, Wainwright K, Miller M, Penzhorn BL, Carreno RA (2006). *Babesia conradae* sp. nov., a small canine *Babesia* identified in California. Vet Parasitol.

[6] García AT (2006). Piroplasma infection in dogs in northern Spain. Vet Parasitol.

[7] Taboada J, Lobetti R, Greene CE (2006). Babesiosis. Infectious diseases of the dog and cat.

[8] Birkenheuer AJ, Levy MG, Breitschwerdt EB (2003). Development and evaluation of a seminested PCR for detection and differentiation of *Babesia gibsoni* (Asian genotype) and *B. canis* DNA in canine blood samples. J Clin Microbiol.

[9] Teal AE, Habura A, Ennis J, Keithly JS, Madison-Antenucci S (2012). A new real time PCR assay for improved detection of the parasite *Babesia microti*. J Clin Microbiol.

[10] Vascellari M, Ravagnan S, Carminato A, Cazzin S, Carli E, Da Rold G (2016). Exposure to vector-borne pathogens in candidate blood donor and free-roaming dogs of northeast Italy. Parasit Vectors.

[11] Paparini A, Senanayake SN, Ryan UM, Irwin PJ (2014). Molecular confirmation of the first autochthonous case of human babesiosis in Australia using a novel primer set for the beta-tubulin gene. Exp Parasitol.

[12] Peleg O, Baneth G, Eyal O, Inbar J, Harrus S (2010). Multiplex real-time qPCR for the detection of *Ehrlichia canis* and *Babesia canis vogeli*. Vet Parasitol.

[13] Matsuu A, Ono S, Ikadai H, Uchide T, Imamura S, Onuma M, et al. Development of a SYBR green real-time polymerase chain reaction assay for quantitative detection of *Babesia gibsoni* (Asian genotype) DNA. J Vet Diagn Invest. 2005;17:569–73. 10.1177/104063870501700608 16475516

[14] Zahler M, Schein E, Rinder H, Gothe R (1998). Characteristic genotypes discriminate between *Babesia canis* isolates of differing vector specificity and pathogenicity to dogs. Parasitol Res.

[15] Kordick SK, Breitschwerdt EB, Hegarty BC, Southwick KL, Colitz CM, Hancock SI (1999). Coinfection with multiple tick-borne pathogens in a Walker Hound kennel in North Carolina. J Clin Microbiol.

[16] Wilson RJ, Williamson DH (1997). Extrachromosomal DNA in the Apicomplexa. Microbiol Mol Biol Rev.

[17] Schreeg ME, Marr HS, Griffith EH, Tarigo JL, Bird DM, Reichard MV (2016). PCR amplification of a multi-copy mitochondrial gene (cox3) improves detection of *Cytauxzoon felis* infection as compared to a ribosomal gene (18S). Vet Parasitol.

[18] Salem GH, Liu X, Johnsrude JD, Dame JB, Roman RG (1999). Development and evaluation of an extra chromosomal DNA-based PCR test for diagnosing bovine babesiosis. Mol Cell Probes.

[19] Bilgic HB, Karagenç T, Shiels B, Tait A, Eren H, Weir W (2010). Evaluation of cytochrome *b* as a sensitive target for PCR based detection of *T. annulata* carrier animals. Vet Parasitol.

[20] Buling A, Criado-Fornelio A, Asenzo G, Benitez D, Barba-Carretero JC, Florin-Christensen M (2007). A quantitative PCR assay for the detection and quantification of *Babesia bovis* and *B. bigemina*. Vet Parasitol.

[21] Haanshuus CG, Mohn SC, Mørch K, Langeland N, Blomberg B, Hanevik K (2013). A novel, single-amplification PCR targeting mitochondrial genome highly sensitive and specific in diagnosing malaria among returned travelers in Bergen, Norway. Malar J.

[22] Isozumi R, Fukui M, Kaneko A, Chan CW, Kawamoto F, Kimura M (2015). Improved detection of malaria cases in island settings of Vanuatu and Kenya by PCR that targets the *Plasmodium* mitochondrial cytochrome *c* oxidase III (cox3) gene. Parasitol Int.

[23] Di Cicco MF, Downey ME, Beeler E, Marr H, Cyrog P, Kidd L (2012). Re-emergence of *Babesia conradae* and effective treatment of infected dogs with atovaquone and azithromycin. Vet Parasitol.

[24] Birkenheuer AJ, Horney B, Bailey M, Scott M, Sherbert B, Catto V (2010). *Babesia microti*-like infections are prevalent in North American foxes. Vet Parasitol.

[25] Birkenheuer AJ, Correa MT, Levy MG, Breitschwerdt EB (2005). Geographic distribution of babesiosis among dogs in the United States and association with dog bites: 150 cases (2000–2003). J Am Vet Med Assoc.

[26] Hikosaka K, Watanabe Y, Tsuji N, Kita K, Kishine H, Arisue N (2010). Divergence of the mitochondrial genome structure in the apicomplexan parasites, *Babesia* and *Theileria*. Mol Biol Evol.

[27] Hikosaka K, Tsuji N, Watanabe Y, Kishine H, Horii T, Igarashi I (2012). Novel type of linear mitochondrial genomes with dual flip-flop inversion system in apicomplexan parasites *Babesia microti* and *Babesia rodhaini*. BMC Genomics.

[28] Schreeg ME, Marr HS, Tarigo JL, Cohn LA, Bird DM, Scholl EH (2016). t al. Mitochondrial genome sequences and structures aid in the resolution of *Piroplasmida* phylogeny. PLoS One.

[29] The Center for Devices and Radiological Health. Statistical guidance on reporting results from studies evaluating diagnostic tests. In: Guidance for Industry and FDA Staff. U.S. Food and Drug Administration. 2007. http://www.fda.gov/RegulatoryInformation/Guidances/ucm071148.htm#6. [Accessed 28 Feb 2017]

[30] Feinstein AR, Cicchetti DV. High agreement but low kappa: I. The problems of two paradoxes. J Clin Epidemiol. 1990;43:543–9. 10.1016/0895-4356(90)90158-l 2348207

[31] Agresti A, Coull BA (1998). Approximate is better than “exact” for interval estimation of binomial proportions. Am Stat.

[32] Yisaschar-Mekuzas Y, Jaffe CL, Pastor J, Cardoso L, Baneth G (2013). Identification of *Babesia* species infecting dogs using reverse line blot hybridization for six canine piroplasms, and evaluation of co-infection by other vector-borne pathogens. Vet Parasitol.

[33] Jefferies R, Ryan UM, Irwin PJ (2007). PCR-RFLP for the detection and differentiation of the canine piroplasm species and its use with filter paper-based technologies. Vet Parasitol.

[34] Wang G, Wormser G, Zhuge J, Villafuerte, Ip D, Zeren C, Fallon J. Utilization of a real-time PCR assay for diagnosis of *Babesia* microti-like infection in clinical practice. Ticks and Tick Borne Dis. 2015; 6:376–382 10.1016/j.ttbdis.2015.03.001 25819568

